# Translational study of the whole transcriptome in rats and genetic polymorphisms in humans identifies *LRP1B* and *VPS13A* as key genes involved in tolerance to cocaine-induced motor disturbances

**DOI:** 10.1038/s41398-020-01050-7

**Published:** 2020-11-06

**Authors:** Florence Vorspan, Romain Icick, Nawel Mekdad, Cindie Courtin, Vanessa Bloch, Frank Bellivier, Jean-Louis Laplanche, Nathalie Prince, Dmitry Pishalin, Cyril Firmo, Corinne Blugeon, Bruno Mégarbane, Cynthia Marie-Claire, Nadia Benturquia

**Affiliations:** 1 Université de Paris, INSERM UMR-S 1144, Optimisation thérapeutique en neuropsychopharmacologie, OTeN, F-75006 Paris, France; 2 Département de Psychiatrie et de Médecine Addictologique, Hôpitaux Lariboisière-Fernand Widal, GHU APHP.Nord–Université de Paris, Paris, F-75010 France; 3 Pharmacie Hospitalière, Hôpitaux Lariboisière-Fernand Widal, GHU APHP.Nord–Université de Paris, Paris, F-75010 France; 4 Département de Biochimie et Biologie Moléculaire, DMU BioGeM, Hôpitaux Lariboisière-Fernand Widal, GHU APHP.Nord–Université de Paris, Paris, F-75010 France; 5 Genomics Core Facility, Institut de Biologie de l’ENS (IBENS), Département de biologie, École normale supérieure, CNRS, INSERM, Université PSL, 75005 Paris, France; 6 Department of Medical and Toxicological Critical Care, Hôpitaux Lariboisière-Fernand Widal, GHU APHP.Nord–Université de Paris, Paris, F-75010 France

**Keywords:** Clinical genetics, Addiction

## Abstract

Motor disturbances strongly increase the burden of cocaine use disorder (CUDs). The objective of our translational study was to identify the genes and biological pathways underlying the tolerance to cocaine-induced motor effects. In a 5-day protocol measuring motor tolerance to cocaine in rats (*N* = 40), modeling the motor response to cocaine in patients, whole-genome RNA sequencing was conducted on the ventral and dorsal striatum to prioritize a genetic association study in 225 patients with severe CUD who underwent thorough phenotypic (cocaine-induced hyperlocomotion, CIH; and cocaine-induced stereotypies, CIS) and genotypic [571,000 polymorphisms (SNPs)] characterization. We provide a comprehensive description of the rat striatal transcriptomic response to cocaine in our paradigm. Repeated vs. acute cocaine binge administration elicited 27 differentially expressed genes in the ventral striatum and two in the dorsal striatum. One gene, *Lrp1b*, was differentially expressed in both regions. In patients, *LRP1B* was significantly associated with both CIS and CIH. CIH was also associated with *VPS13A*, a gene involved in a severe neurological disorder characterized by hyperkinetic movements. The *LRP1B* minor allele rs7568970 had a significant protective effect against CIS (558 SNPs, Bonferroni-corrected *p* = 0.02) that resisted adjustment for confounding factors, including the amount of cocaine use (adjusted beta = −0.965 and −2.35 for heterozygotes and homozygotes, respectively, *p* < 0.01). Using hypothesis-free prioritization of candidate genes along with thorough methodology in both the preclinical and human analysis pipelines, we provide reliable evidence that *LRP1B* and *VPS13A* are involved in the motor tolerance to cocaine in CUD patients, in line with their known pathophysiology.

## Introduction

Cocaine use disorder (CUD) is a severe condition associated with both mortality^1^ and morbidity^2,3^. Furthermore, this disorder places a large burden on individuals, families and society^4^. Most studies that have attempted to identify therapeutic strategies for CUD have shown limited effectiveness, whether they used pharmacological^5^ or psychosocial^6^ interventions. One of the most burdensome features of CUD is motor disturbances, which are likely involved in the legal consequences of the disorder^7^. Agitation is indeed the second most frequent motive for patients’ referral to the emergency department, after anxiety but before chest pain^8^. Symptoms of motor disturbances are usually considered to represent the transient acute dopaminergic effect of the drug on central motor structures, especially the striatum, as well as the prefrontal cortex. The striatum is thus expected to play a key role in the tolerance to cocaine-induced motor effects. This structure is located in the basal ganglia and is divided into dorsal and ventral regions. The dorsal striatum regulates movement and cognition^9,10^, whereas the ventral striatum modulates reward and emotion^11–13^. In rodent models, a shift from ventral to dorsal activation during reward processes has been described as a key mechanism in tolerance to the rewarding effects of chronic substance use, from goal-directed behavior to compulsive drug use^14,15^.

Cocaine-induced transient motor symptoms mostly consist of either agitated/aggressive behavior or repetitive, purposeless, stereotyped movements (cocaine-induced stereotypies, CIS). Using the definition from a validated rating questionnaire (Scale for Assessment of Positive Symptoms—Cocaine-Induced Psychosis, SAPS-CIP)^16^ in two independent clinical samples, the prevalence of agitation was found to be 41–45% and that of CIS was 58–74%^17,18^. Thus, a substantial number of patients may be tolerant to these symptoms despite chronic cocaine use, suggesting the existence of genetic susceptibility. The acquisition of this form of tolerance may not occur at the same time as the acquisition of the Diagnostic and Statistical Manual (DSM) criterion of tolerance, which mostly applies to the rewarding effects of the drug, as was evidenced in a longitudinal study of heavy cocaine users^19^. Motor tolerance is far less studied than is CUD. Large-scale GWAS of cocaine-induced agitation and CIS are thus warranted; however, such studies would require in-depth phenotyping and would need to take into account dose-effect relationships, which is rarely possible in large GWAS-size samples. Preclinical models can overcome these limitations by strictly controlling for cocaine exposure and prioritizing candidate genes/pathways to be tested in humans.

In rodents, binge rather than intermittent cocaine administration is associated with motor tolerance, as measured by locomotor activity^20–22^ or stereotypies^23^, providing preliminary insights into the pathophysiology of these motor phenomena. To further identify genes and biological pathways that underlie motor tolerance to cocaine, we chose to perform a translational study. Whole-transcriptome analysis focusing on the two previously described crucial striatal rat brain regions was conducted to identify the genes associated with the acquisition of motor tolerance in a specific preclinical behavioral paradigm. Differentially expressed genes (DEGs) were further tested for their association with a quantitative measurement of hyperlocomotion and stereotypies, the corresponding motor phenotypes, in patients with severe CUD.

## Materials and methods

An overview of the current study design is provided in Supplementary Fig. 1.

See the Supplementary methods file for details.

### Preclinical study

#### Animals

Male Sprague-Dawley rats (Charles-River, France), weighing 250–300 g at the start of the experiment were housed in an environment maintained at 20 ± 1 °C with controlled humidity and on a 12/12-h light/dark cycle (light at 8 a.m.). Food and water were provided ad libitum. Animals were acclimated to the animal housing facility for 1 week and were handled daily. Animals were treated in accordance with the European Communities Council Directive (86/609/EEC), French law, and standard ethical guidelines under the control of the institutional ethical committee of Paris Descartes University (N° CEEA34. NB.127.12). The number of animals used and their suffering were minimized in all experiments.

#### Chemicals

Cocaine hydrochloride (Francopia, Anthony, France) was dissolved in sterile saline solution (0.9% (w/v) NaCl). Rats received 20 mg/kg cocaine in 1 ml/kg of body weight by intraperitoneal (i.p.) route.

#### Motor behavior

**Fig. 1:**
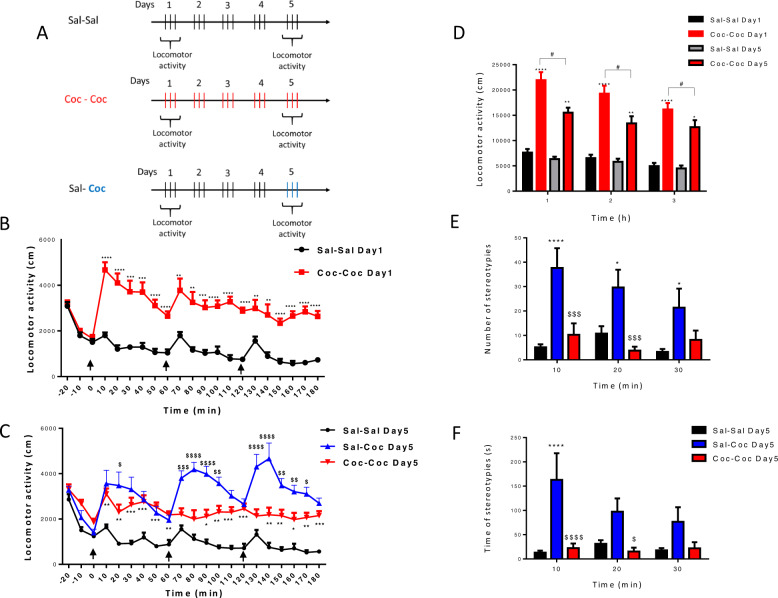
Preclinical study. **a** Study design of cocaine binge administration. Rats were randomly divided into three groups (saline–saline, cocaine–cocaine, and saline–cocaine). Rats received 20 mg/ kg cocaine hydrochloride (red and blue bars) or saline (black bars) i.p. three times a day at 10:00, 11:00 a.m., and 12:00 p.m. for five successive days. Locomotor activity expressed as the total distance traveled during 3 h every 10 min on Day 1 (**b**) on Day 5 (**c**), the time of cocaine or saline administration is indicated by black arrows. Locomotor activity expressed as the total distance traveled during 3 h every hour on Day 1 and on Day 5 (**d**). Number of stereotypies (**e**) and time of stereotypies (**f**) every 10 min during 30 min after the last administration on Day 5. Values are expressed as mean ± SEM (*n* = 8–12 for each group). Comparisons were performed using two-way repeated measures followed by post-tests with Bonferroni correction. Cocaine–Cocaine Day 1 versus Saline–Saline Day 1; Cocaine–Cocaine Day 5 versus Saline–Saline Day 5: *p < 0.05, ***p < 0.001, and ****p < 0.0001; Cocaine–Cocaine Day 1 versus Cocaine–Cocaine Day 5: ^#^*p* < 0.05, ^###^*p* < 0.001 and Cocaine–Cocaine Day 5 versus Saline–Cocaine Day 5: ^$^*p* < 0.05, ^$$$^*p* < 0.001, ^$$$$^*p* < 0.0001.

Locomotor activity and stereotypies were measured on the lighting cycle as previously described^24^. Rat locomotor activity was measured in an open field (OPF) consisting of an enclosed white Plexiglas chamber open at the top, divided into four equal-sized areas (50 × 50 × 35 cm), and maintained under low illumination (10 lux). Twenty-four hours prior to the experiment, rats received one habituation session for 10 min. At the experiment time, the first administration was preceded by habituation to OPF lasting 30 min. Immediately after each treatment administration (three times a day, 1 h apart at 10:00, 11:00 a.m. and 12:00 p.m. for the repeated cocaine profile), according to the study design (Fig. 1a), one rat was placed in each area and recorded during 1 h according to the study design. The chamber was fitted with an infrared floor connected to a miniature overhead infrared video-camera and a PC that used automated video-tracking software (ViewPoint, VideoTrack, Lyon, France) to determine rat horizontal locomotor activity (traveled distance in cm). Stereotypies (number and time) were recorded using an ethological keyboard: stereotyped head weaving (repetitive turning of the head from side to side), stereotyped circular head movements, stereotyped licking (repeatedly licking objects other than oneself such as the Plexiglas chamber), other stereotyped compulsive movements (any repetitive movement that does not fall under other categories of stereotyped behavior).

#### RNA extraction and library preparation

Quantification of total RNA was performed using a NanoDrop™ One spectrophotometer (Nanodrop Technologies Inc., Wilmington, DE, USA). Rats were sacrificed 24 h after the last cocaine injection. Total RNA was extracted, from 3 pools of nucleus accumbens or caudate putamen of 2 rats, with an RNeasy Qiagen mini kit according to the lipid tissue protocol of the manufacturer (France). Messenger RNAs, from 3 pools of 2 dorsal or ventral striatum from saline–saline (Sal–Sal) Day 5, saline–cocaine (Sal–Coc) Day 5 or cocaine–cocaine (Coc–Coc) Day 5-treated rats, were purified from 500 ng of total RNA using oligo(dT). Samples were randomized and strand-specific RNA-seq libraries were prepared with a TruSeq Stranded mRNA kit (Illumina, USA). Next, 75 bp single read sequencing was performed on a NextSeq 500 instrument (Illumina, USA). A mean of 53 ± 4 million reads passing the Illumina quality filter was obtained for each of the samples.

### Data analysis

#### Behavioral study

Analysis of locomotor activity was performed with Student’s *t-*tests or two-way analysis of variance (ANOVA) followed by appropriate post hoc tests for multiple comparisons with Bonferroni correction using GraphPad Prism version 7.00 (GraphPad Software, USA).

#### RNA-Seq study

Before mapping, poly-N read tails were trimmed, reads ≤40 bases were removed, and reads with a quality mean ≤30 were discarded. Alignments from reads mapping more than once to the reference genome were removed using the Java version of SAM tools (6). Statistical treatments and differential expression analyses were performed using DESeq 1.8.3^25^. Analyses were performed using the Eoulsan pipeline^26^. The *Rattus norvegicus* reference genome from Ensembl version 84 was used for both read alignment (STAR version 2.4.0k^27^) and gene expression analysis (GFF3 genome annotation). The raw data are available on the GEO repository (www.ncbi.nlm.nih.gov/geo/) under accession numbers GSE134154 and GSE134107. Pathway enrichment analyses were performed using the Kyoto Encyclopedia of Genes and Genomes (KEGG) with the WEB-based GEne SeT AnaLysis Toolkit, WebGestalt (http://www.webgestalt.org/)^28^. The significance threshold was set at 0.05 using false discovery rate (FDR) correction.

### Clinical study

#### Sample selection and clinical assessment

Patients > 18 years of age seeking treatment for any substance use disorders (SUD) were consecutively recruited according to a multicenter protocol aimed at characterizing the phenotypic and genotypic architecture of severe CUDs^29^ (clinical trial number NCT01569347). All included participants provided written informed consent for both the clinical and genetic assessments. Study protocols and analyses were approved by ad hoc ethics boards (CPP Ile-de-France IV and INSERM institutional review board IRB00003888). All clinical data were obtained through a single, face-to-face, semi-structured interview^30^ with a trained M.D. or psychologist, thus collecting patients’ self-reported clinical history. As part of the extensive phenotypic characterization, we used two 5-point clinician-rated subscales of the SAPS-CIP: the agitation subscale for CIH and the compulsive movement subscale for CIS, which were translated and back-translated to obtain French versions^31^. To ensure that only patients with chronic cocaine use who had enough cocaine exposure or develop a tolerance to the rewarding effect would be included, CIH and CIS were evaluated in only those participants who met the DSM-IV tolerance criterion.

#### DNA sampling

DNA was extracted from whole blood collected from participants using a Maxwell 16 PROMEGA extractor (Promega, France). Participants were genotyped using the Infinium PsychChip array (Illumina, USA) processed in two stages (2015 and 2017) by Integragen SA (France).

### Genetic analyses: single nucleotide polymorphisms

*PLINK* software^28^ was first used to merge the two raw genotype files generated by the two waves of genotyping, based on biallelic markers, yielding a starting pool of 566,932 markers. Then, *PLINK* was used for quality control (QC), based on a consensus procedure for ancestry, relatedness, and genetic discrepancies^32^. The final study sample thus comprised 337 individuals with a mean 99.831% genotyping rate across 260,853 markers. The main procedure in the preclinical study elicited 28 genes for the candidate association study (two, *Zfp871* and the unidentified LOC100912852, are absent from the human genome). All 26 were available for gene-based tests. QC and minor allele frequency filters left 558 SNPs from 24 genes for SNP-based tests (among 1510 markers from 26 genes, one of which had only SNPs with minor allele frequencies <5% and one of which was on chromosome X), yielding a *P*-value threshold for 558 tests in two phenotypes of 0.0000448.

### Genetic analyses: copy number variants

We followed the pipeline used in a recent study^33^, starting from the Illumina® final report file generated by *GenomeStudio v.2*, which involved two detection programs, *PennCNV*^34^ and *QuantiSNP*^35^, optimized in a combined algorithm that allows for a thorough quality check. Both programs are based on the hidden Markov chain model but show different sensitivities and specificities.

### Data analysis

#### Phenotyping

Continuous variables were described by means (standard deviation, SD) or medians (interquartile range, IQR) depending on their distribution, and qualitative variables are described by absolute counts and frequencies. Nonparametric tests were used to identify the clinical and sociodemographic factors associated with CIH and CIS and were further incorporated into the ordinal regression model.

#### Genetics

The candidate genes identified in the preclinical study were searched for copy number variants (CNVs) through the genome-wide list constructed by the detection pipeline in the clinical sample. The identified CNVs were further tested for associations with CIH and CIS severity based on their frequency, size, or type (duplication vs. deletion) in a subsample of Caucasian ancestry. Gene-based (adaptive permutation followed by Bonferroni correction) and SNP-based (Bonferroni correction) analyses were performed using PLINK ad hoc functions, with α = 0.05 after correction as a significance threshold. Genetic associations were further tested by ordinal regression with CIH and CIS scores as dependent variables in *R* through the ‘ordinal’ package, which allowed for adjustment on potential confounders.

In silico functional analyses were performed online using the Functional Mapping and Annotation of Genome-Wide Association Studies (FUMA) platform^36^ and the 3D Genome Browser^37^ to identify topologically-associated domains^38^. In case more than two genes were associated with a given phenotype, the Kyoto Encyclopedia of Genes and Genomes (KEGG), PANTHER and Biological General Repository for Interaction Dataset (BioGRID) database were searched for enriched pathways and all disease/phenotype-based repositories for enriched gene sets using the WEB-based GEne SeT AnaLysis Toolkit, WebGestalt (http://www.webgestalt.org/)^28^. The STRING database (Protein–Protein Interaction Networks Functional Enrichment Analysis, https://string-db.org/)^32^ was also searched for gene × gene and protein × protein interactions. The significance threshold was set at 0.05 using false discovery rate (FDR) correction.

## Results

### Preclinical behavioral study

#### Locomotor activity

Acute cocaine-induced hyperlocomotion in comparison to the control on Day 1 (Fig. 1b). A two-way ANOVA showed significant treatment × time interaction (F(18,324) = 2.863, *p* < 0.0001), with significant time (F(18,324) = 7.103, *p* < 0.0001) and treatment effects (F(1,18) = 60.06, *p* < 0.0001). Post hoc analyses showed significant increase in locomotion in the Coc–Coc Day 1 group compared with Sal–Sal Day 1 lasting at least 1 h after each 3 cocaine administration. On Day 5 (Fig. 1c), a two-way ANOVA repeated measures on locomotor activity every 10 min showed significant treatment × time interaction (F(36,486) = 4.662, *p* < 0.0001), with significant time (F(18,486) = 5.820, *p* < 0.0001) and treatment effects (F(2,27) = 32.580, *p* < 0.0001). Bonferroni’s post hoc analyses showed significant increase in locomotion in the Coc–Coc Day 5-treated rats compared with Sal–Coc Day 5 group. On Day 5 vs. Day 1 (Fig. 1d), a two-way repeated-measure ANOVA on cumulative locomotor activity every hour showed a significant treatment × time interaction (F_(10,112)_ = 2.409, *p* = 0.0124) with significant time (F_(2,112)_ = 4.072, *p* = 0.0196) and treatment effects (F_(5,56)_ = 32.480, *p* < 0.0001). Bonferroni’s post hoc analyses showed a significant decrease in locomotion (tolerance) in the Coc–Coc-treated rats on Day 5 compared with day 1 at each hour (*p* < 0.05 for each hour).

#### Stereotypies

On Day 5, after the last administration, a two-way repeated measures ANOVA every 10 min for 30 min showed a treatment effect on the number of stereotypies (F_(2,25)_ = 11.510, *p* = 0.0003) (Fig. 1c) and an interaction of time × treatment on the time of stereotypies (F_(4,50)_ = 3.694, *p* = 0.0104) and treatment effect (F_(2.25)_ = 8.343, *p* = 0.0017) (Fig. 1d). Bonferroni’s post hoc analyses showed significant tolerance for the number and time of stereotypies during the 20 min after the last cocaine administration.

### RNA-seq analysis of differentially expressed genes in the striatum

#### Ventral striatum

**Fig. 2:**
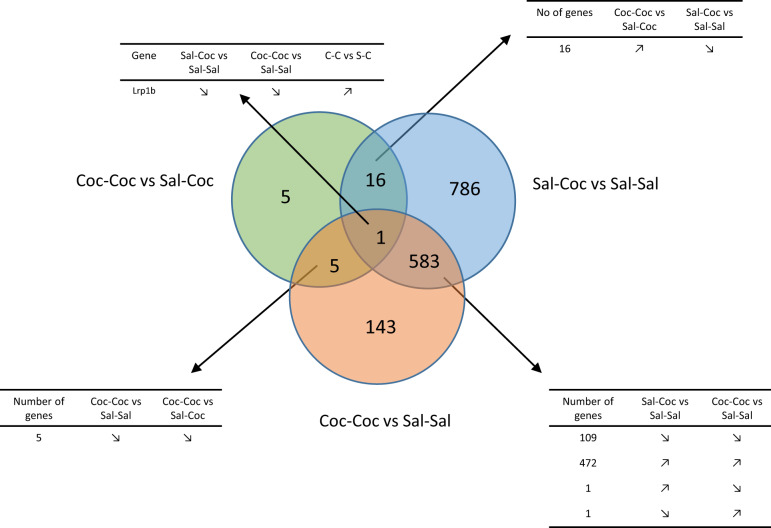
Preclinical study. Differentially expressed genes (DEGs) in the ventral striatum. Venn diagram of the DEGs in the ventral striatum. Modulations of the common DEGs in the three tested comparisons are detailed.

A total of 22,670 genes were detected. When comparing the Coc–Coc and Sal–Coc vs. Sal–Sal conditions, we identified 733 and 1386 DEGs, respectively, thus evidencing a broad transcriptomic response to cocaine administration in the ventral striatum (Fig. 2). The 1386 DEG in the Sal–Coc vs. Sal–Sal condition belonged to 33 significantly enriched pathways (the top 20 are presented in Supplementary Table 1). The 733 DEG in the Coc–Coc vs. Sal–Sal condition belonged to four significantly enriched pathways (Supplementary Table 1). Twenty-seven (27) DEGs were identified in the Coc–Coc vs. Sal–Coc comparison, as presented in Supplementary Table 2. As seen in Fig. 2, one gene, *Lrp1b*, was at the crossroads of cocaine-induced transcriptomic response in the ventral striatum regardless of administration condition: upregulated in the Coc–Coc vs. Sal–Coc condition (2.69-fold) and downregulated in both the Sal–Coc and Coc–Coc vs. Sal–Sal conditions (0.21- and 0.58-fold, respectively). The 27 DEG of the Coc–Coc vs. Sal–Coc condition belonged to four significantly enriched pathways: neuroactive ligand–receptor interaction, MAPK signaling pathway, amyotrophic lateral sclerosis (ALS), and cocaine addiction (Supplementary Table 1).

#### Dorsal striatum

**Fig. 3:**
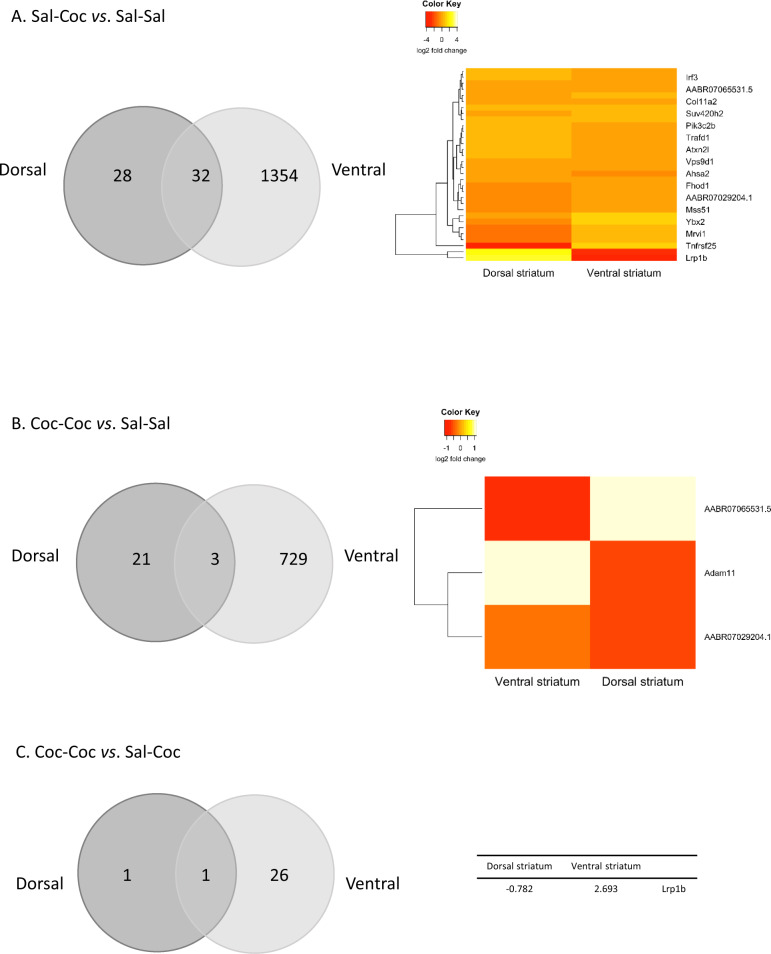
Preclinical study. Differentially expressed genes (DEGs) in the ventral and dorsal striatum in the three experimental conditions. **a** Venn diagram of the DEGs in the Sal–Coc vs. Sal–Sal comparison in the ventral and the dorsal striatum. Heatmaps of the log2 fold change of the common DEGs are detailed. **b** Venn diagram of the DEGs in the Coc–Coc vs. Sal–Sal comparison in the ventral and the dorsal striatum. Heatmaps of the log2 fold change of the common DEGs are detailed. **c** Venn diagram of the DEGs in the Coc–Coc vs. Sal–Coc comparison in the ventral and the dorsal striatum. Log2 fold change of the common DEG are shown.

In total, 32,403 genes were detected, and the transcriptional effects of cocaine (in both the Coc–Coc and Sal–Coc groups) were more specific than in the ventral striatum (Supplementary Fig. 2). Interestingly, among the DEGs in the Coc–Coc vs. Sal–Coc comparison, *Lrp1b* was the only DEG shared between the dorsal and ventral striatum, and it displayed opposite regulation between the two structures (Fig. 3). The 60 DEG of the Sal–Coc vs. Sal–Sal condition belonged to one significantly enriched pathway (Lysine degradation, Supplementary Table 1). The Coc–Coc vs. Sal–Sal and the Coc–Coc vs. Sal–Coc conditions did not elicit any significantly enriched pathway.

RT-qPCR performed on the same samples confirmed the modulation of *Lrp1b* in the ventral but not in the dorsal striatum (Supplementary Fig. 3). The complete list of DEGs in the two studied structures are presented in Supplementary Table 3.

### Genetic association studies in patients

Among the 418 genotyped subjects, 393 (94%) passed QC, and 325 (70%) met the DSM-IV criterion “tolerance to cocaine”. Two hundred twenty-five (225) subjects had valid CIH and CIS data, which were distributed as follows. For CIH, the mean was 1.6 (SD = 1.8), and the median was 1 [IQR = 1–3, 104 (46%) patients with a score ≥2 indicating clinical significance]. For CIS, the mean was 2.3 (SD = 1.5), and the median was 3 [IQR = 1–3, 159 (71%) patients with clinically significant CIS] (Supplementary Table 4). This suggests that the patients had overall higher CIS than CIH scores. Gene-based tests were performed on 26 genes using up to 100,000 permutations. SNP-based tests were performed on 558 markers from 24 genes. Among the 1362 CNVs that passed QC in 334 individuals (Supplementary Fig. 4), none were located in any of our candidate genes. Thus, we did not perform any additional CNV-based analyses.

#### Locomotor activity (CIH)

**Fig. 4:**
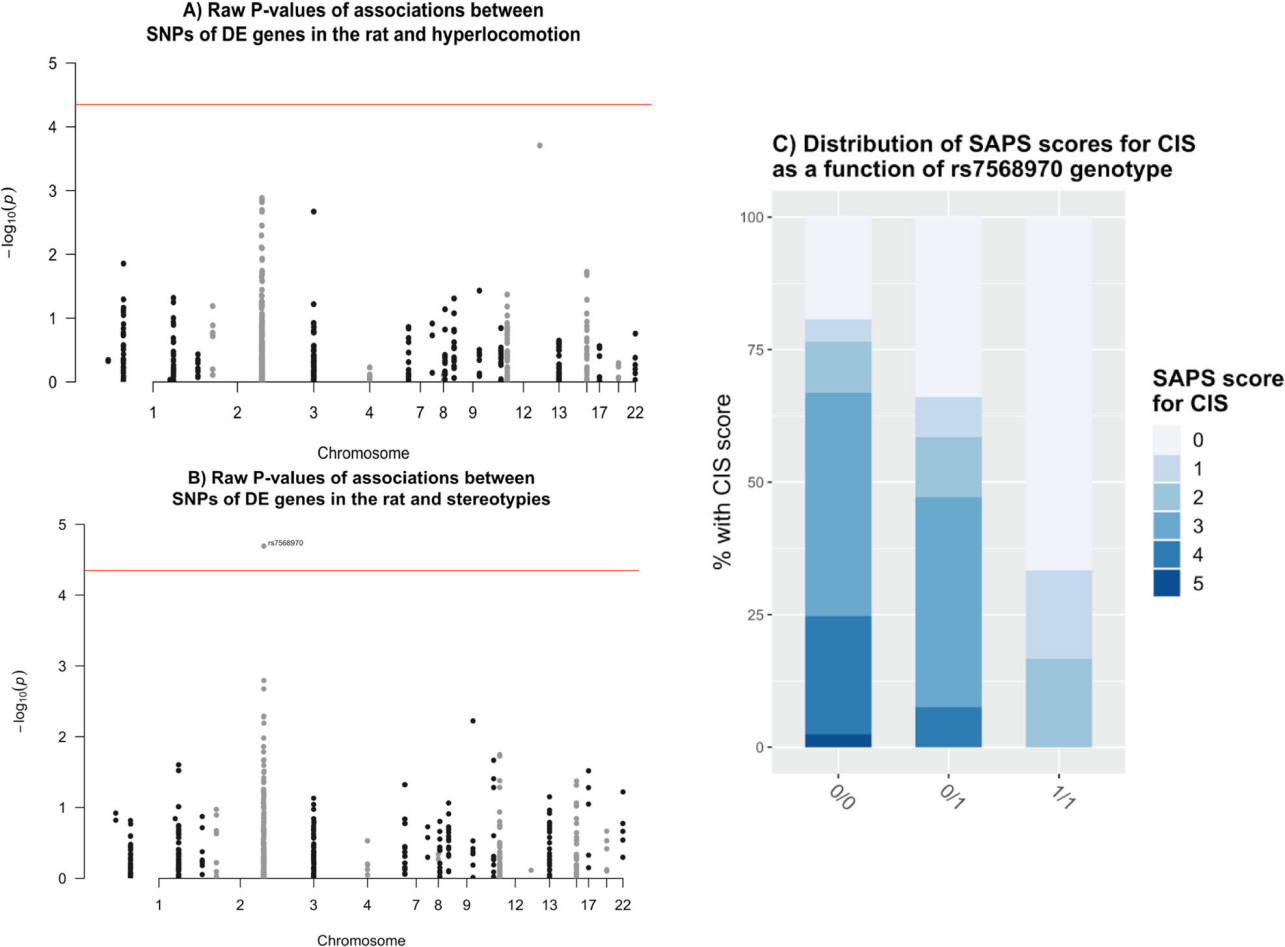
Human genetics. **a** Manhattan plot of raw *p*-values for associations between 558 markers and cocaine-induced hyperlocomotion (CIH). The red line represents the significance threshold after Bonferroni correction. **b** Manhattan plot of raw *p*-values for associations between 558 markers and cocaine-induced stereotypies (CIS). The red line represents the significance threshold after Bonferroni correction. **c** CIS as a function of *LRP1B* rs7568970 genotype. G is the minor allele. SAPS-CIP, Scale for Assessment of Positive Symptoms – Cocaine-Induced Psychosis.

CIH was associated with *NTS* (*p*_corr_ = 0.000772), *VPS13A* (*p*_corr_ = 0.003024), *LRP1B* (*p*_corr_ = 0.00301), *LRRC7* (*p*_corr_ = 0.00301), *CACNA1E* (*p*_corr_ = 0.00301), *GRIN2B* (*p*_corr_ = 0.00301), *ZBTB20* (*p*_corr_ = 0.00301), *CDH8* (*p*_corr_ = 0.00301), and *ELAVL2* (*p*_corr_ = 0.00301). There was no significant association at the SNP level (Fig. 4a, top SNP = *NTS* rs10863088, *p*_corr_ = 0.2272).

#### Stereotypies (CIS)

Gene-based tests identified significant associations between CIS and *LRP1B* (five SNPs, *p*_corr_ = 0.00312). At the SNP level, a significant association was found between CIS score and rs7568970 in *LRP1B* (*p*_corr_ = 0.02269) (Fig. 4b). Furthermore, there was a clear inverse relationship between *LRP1B* minor allele dosage and CIS score (Fig. 4c). Ordinal logistic regression confirmed the association between *LRP1B* rs7568970 and CIS score (Table 1). The association remained after adjusting for age, sex, DSM-IV lifetime benzodiazepine dependence, and heaviest self-reported monthly cocaine use (*z*-value = −3.08, *p* = 0.002 for A/G genotypes and *z*-value = −2.31, *p* = 0.0066 for G/G genotypes). After grouping by low (0–1), moderate (2–3), and severe (4–5) CIS, carrying one copy of the rs7568970 minor allele conferred a 62% lower risk of belonging to the next more severe group (moderate vs. low and high vs. moderate). The risk was 94% lower for those carrying two copies. Supplementary Table 5 summarizes the results of linear regressions on CIS and CIH.

**Table 1:** Human genetics. Ordinal regression of rs75689870 and clinical variables with cocaine-induced stereotypies as a dependent variable.

	Estimate	Std. error	*z-*value	*p*-value
*LRP1B* rs7568970 A/G	−0.965	0.285	−3.39	0.000712***
*LRP1B* rs7568970 G/G	−2.35	0.848	−2.77	0.005572**
Lifetime sedative use disorder (present vs. absent)	0.228	0.246	0.929	0.353086
Heaviest monthly cocaine use (0–30 days/month)	0.0279	0.014	1.99	0.047061*
Age	−0.0195	0.013	−1.5	0.132576
Gender (men vs. women)	0.504	0.299	1.68	0.092541

*p < 0.05, **p < 0.01, and ***p < 0.001.

#### In silico functional analysis

We further searched for functional significance of (i) *LRP1B* SNP rs7568970 and (ii) the list of genes associated with CIH.

rs7568970 is an intronic variant in the large *LRP1B* gene (ID ENSG00000168702.1, chr. 2:140988992–142889270, 1.9+ mega base pairs, 91 exons), which encodes the LDL Receptor-Related Protein 1B, a putative tumor suppressor and member of the low-density lipoprotein (LDL) receptor family that is highly expressed in the human brain. There were 15 other intronic SNPs in significant LD with rs7568970 (*r*^2^ > 0.6), which may have a potentially greater impact on gene function. None of them was significantly associated with any brain eQTL nor any mQTL (other than during pregnancy) nor any topologically-associated domain. All SNPs could be scored for chromatin/DNA-enzymes interactions in the regulomeDB database. Amongst them, rs62166486 was scored 3a (transcription factor binding + any motif + DNase peak), and rs16846586 4 (transcription factor binding + DNase peak). Finally, the Phred scores for the deleteriousness of these SNPs according to the combined annotation-dependent depletion (CADD) database^39^ ranged from 0.078 to 16.33. Four SNPs, rs16855067, rs72857311, rs62166498, and rs62166499, had CADD scores ≥10, indicating that these are predicted to be the 10% most deleterious substitutions that you can do to the human genome.

The genes associated with CIH did not yield any significant KEGG or PANTHER pathway (top pathway = “cocaine addiction” in KEGG, *p*-FDR = 1) or any significant interactions from protein networks (*p*-FDR = 0.7623). When searching the “Human phenotype ontology” database, four genes were flagged as belonging to the “Developmental regression” pathway (HP:0002376; *GRIN2B*, *LYST*, *VPS13A*, and *ZBTB20*; *p*-FDR = 0.048). Finally, STRING reported one interaction between *LRRC7* and *GRIN2B* involving gene coexpression (*p*-FDR = 0.0015).

## Discussion

In the present translational study, we first performed a transcriptome-wide analysis focused on brain regions that are crucial to the acquisition of tolerance to hyperlocomotion and stereotypies during a repeated “binge” cocaine administration protocol in rats. The association of DEGs with the relevant motor phenotypes in humans, CIH and CIS, was further tested in a well-characterized clinical sample. The main findings were as follows: (1) in the ventral striatum, 27 genes were differentially expressed between acute and repeated cocaine administration (Sal–Coc vs. Coc–Coc); (2) one of those genes, *Lrp1b*, was differentially modulated in all experimental conditions; (3) accordingly, nine of the 26 DEGs identified in the preclinical study were significantly associated with CIH in patients. (4) One gene, *LRP1B*, was associated with both CIH and CIS. A tag marker of this gene, rs7568970, was further associated with a lower intensity of (i.e., tolerance to) CIS in humans with a strong effect size in the same direction as in the preclinical study, and this association resisted adjustment for confounders.

In our preclinical experiments, motor tolerance has been developed as previously showed^14^ and transcriptomic changes were, overall, more pronounced with single cocaine administration than with repeated cocaine binge in both the ventral and dorsal striatum. Differences between single and repeated cocaine exposure have been previously reported, but the results were mixed, depending on the candidate gene that was investigated^40,41^. Furthermore, it was striking that in the single binge cocaine groups, all 32 genes that were differentially expressed in both striatal regions showed opposite regulation (Fig. 3). These region-specific, early gene regulation patterns could participate in a shift from the activation of the ventral striatum to the activation of the dorsal striatum in response to cocaine^42^. We could not find any published study reporting such a ventral/dorsal striatum imbalance in human subjects with cocaine dependence. However, available evidence suggests that healthy controls show superior activation of the ventral striatum in response to an acute psychostimulant intake^43^ while individuals with cocaine dependence have repeatedly shown a preferential activation of the dorsal striatum^44–46^. A dysfunctional activation of the ventral striatum has also been reported in cocaine dependence^47^, which could represent an intermediate state. Thus, there is suggestive evidence of a shift from ventral to dorsal striatal activation during the acquisition of cocaine dependence in humans, but more efforts should be deployed to better characterize striatal dysfunction along the different stages of cocaine use. The closest preclinical study we could find is an unbiased assessment of gene regulation across various time points of cocaine self-administration—short and long-term withdrawal^48^. They showed that each gene list is unique for a pattern within a brain region (dorsal and ventral striatum), suggesting that the targets of these predicted regulators change depending on cocaine history and re-exposure paradigm. However, that study did not compare the respective activation of set of genes in the dorsal versus ventral striatum during the various stages of cocaine tolerance acquisition, which further highlights the interest of our tolerance-inducing protocol. The observation of a differential activation between the ventral and the dorsal striatum, suggestive of a shift that could occur during the acquisition of the addictive disorder, has been observed for the rewarding effects as a function of substance use history. For example in alcohol use disorder, at least one fMRI study compared the activation of ventral versus dorsal striatum in a cue-induced paradigm in social drinkers (who activated more the ventral striatum) compared to heavy drinkers (who activated more the dorsal striatum). Of note in that study, most heavy alcohol drinkers qualified for DSM-IV alcohol dependence^15^. Interestingly, similar patterns of striatal activation have been reported in cannabis use vs. dependence^49^. Our study is the first one to suggest that the variability to the acquisition of the tolerance to cocaine-induced motor disturbances could also be characterized by a shift in the activation of specific sets of genes from the ventral to the dorsal striatum, and that genetic polymorphisms could mediate this between-subjects variability.

A key finding of our study concerns the *Lrp1b* gene. *Lrp1b* was found to be inversely regulated by repeated *vs*. single cocaine administration in both the dorsal and ventral striatum. In the ventral striatum, *Lrp1b* was the only gene with significantly differential expression in all three tested comparisons. Strikingly, *LRP1B* was also associated with CIS in the human genetic association study and could represent a tag marker for two SNPs (not genotyped here) among the top 1.1–1.3% of deleterious variants in the human genome, according to CADD standards (rs62166498, 932 bp upstream, LD = 1, CADD = 15.66; rs62166499, 920 bp upstream, LD = 1, CADD = 17.85). In human tissues, *LRP1B* is highly expressed in the brain, skeletal muscles, thyroid, and adipocytes (GTEx Analysis Release V8). This expression pattern is broader than that observed in mice, where the expression of *LRP1B* is restricted to the brain^50^. The LRP1B protein is involved in lipid metabolism and in neurodevelopment through cell growth and migration^51^. To the best of our knowledge, there is only one previous report of the potential modulation of *LRP1B* in human addiction. A decrease in the DNA methylation of the *LRP1B* promoter was found in blood cells from patients stabilized at high daily methadone doses compared with patients stabilized at low doses^52^. Interestingly, in that previously published sample^53^, a high methadone dose was significantly associated with lifetime cocaine dependence.

The second robust finding concerns several interesting results on the set of nine genes associated with CIH. First, all those genes are highly and predominantly expressed in the human brain. Most of their proteins are involved in plasma membrane rearrangement, consistent with some of the key mechanisms associated with non-motor aspects of tolerance to cocaine, including changes in receptor density^54–56^, dendritic growth, and synaptic plasticity (for reviews, see refs. ^57,58^). Second, *CACNA1E* encodes the Calcium Voltage-Gated Channel Subunit Alpha1 E. Calcium channel modulation has been repeatedly associated with psychostimulant-induced locomotor activity (e.g., refs. ^59,60^). More specifically, *Cacna1e*^*−/−*^ mice show a complete loss of acute locomotor response to cocaine^61^. Third, some *VPS13A* mutations can cause a severe neurological condition, chorea-acanthocytosis, which is characterized by abnormal motor behavior^62^ and marked loss of striatal volume^63^. In the current preclinical study, *Vps13a* was the only DEG other than *Lrp1b* to be downregulated in the ventral striatum and upregulated in the dorsal striatum in the Sal–Coc vs. Sal–Sal comparison (Supplementary Table 2). Alterations in striatal volume and function have been reported in CUD^64,65^. *VPS13A* has been associated with nicotine dependence^66^, but not CUD, and notably, the association between CIS and *VPS13A* persisted after adjusting for smoking intensity in our sample (data not shown). Fourth, in mice, cocaine elicited increased Neurotensin expression in the ventral striatum^67^, but deletion of the *Nts* gene had no effect on locomotion^68^. Finally, although less specific to motor disturbances, *GRIN2B* has been associated with cocaine dependence at both the genetic and transcriptomic levels, in rodents^69^ and in patients^70^, and thus, it is a possible top candidate gene for cocaine misuse according to a recent meta-analysis of genome-wide expression data^71^. We were unable to find any published report of associations between the other CIH-associated genes and either motor function or cocaine response.

At the clinical level, CIS was significantly associated with the heaviest self-reported cocaine use. This highlights the relative specificity of this measure to the direct motor responses to cocaine, which most likely occur through over-activation of the nigrostriatal dopaminergic pathway. In that regard, the CIH measure in humans elicited broader genetic associations, possibly because this score captured motor symptoms of nervousness or aggression, which can be found in several psychiatric conditions and are thus less specific than CIS^72,73^.

One strength of our study is that we used whole-genome transcriptional results obtained in a rat model of motor tolerance to identify genetic variants of interest in patients seeking treatment for cocaine dependence who were thoroughly assessed for cocaine-induced motor behavior. The preclinical study was region-specific within the striatum and compared single vs. repeated cocaine exposure. In the human sample, we performed extensive phenotypic characterization and thorough genetic QC. Moreover, the regional plot indicated very good coverage of the area (Supplementary Fig. 5) rather than an isolated and possibly spurious hit. However, our study also presents limitations. In our study, the rats were injected by experimenter which allows to see only the genes regulated by the effects of cocaine inducing tolerance but certainly does not take into account the self-administration factor from the human study.

One caveat for our study is that we could not perform peripheral transcriptional analysis in humans since RNA samples were not collected in this cohort. Nevertheless, the present transcriptomic/genetic exploratory approach yielded results in humans that were congruent with the results in rats, suggesting that our significant findings are unlikely to be false positives. In addition, the assessment of motor behavior in humans was retrospective, based on the estimated lifetime worst period of cocaine use, and was thus subject to recall bias. This bias was reduced by the use of a standardized questionnaire to assess motor phenotypes^16^. Finally, although we have gathered important preclinical evidence used to drive a genetic association study with consistent results, we did not circle back to an animal model with modulated *Lrp1b* activity or expressing the rs7568970 SNP. This could leverage the mechanisms of the associations presented in the current study, however, such protocols will require extended time and funding that were not available at the time of the study.

In conclusion, the translational approach presented in this study identified DEGs in a hypothesis-free whole-genome expression rodent model as a first step to prioritize candidate genes to conduct an association study in patients. Our results mainly suggest that *LRP1B* is strongly involved in striatal neuroadaptation to repeated cocaine-exposure-induced motor behavior, as it was associated with both CIH and CIS. Furthermore, the two DEGs upregulated in the dorsal striatum and downregulated in the ventral striatum (*Lrp1b* and *Vps13a*) in the rat Sal–Coc vs. Sal–Sal comparison displayed significant associations with CIS and CIH in humans, respectively. Our results support a translational and multimodal approach to studying complex disorders such as addiction.

## Supplementary information

Supplementary Figure 1

Supplementary Figure 2

Supplementary Figure 3

Supplementary Figure 4

Supplementary Figure 5

Supplementary Table 1

Supplementary Table 2

Supplementary Table 3

Supplementary Table 4

Supplementary Table 5

Supplementary materials
