# Supplementary figures and images for: Translational study of the whole transcriptome in rats and genetic polymorphisms in humans identifies LRP1B and VPS13A as key genes involved in tolerance to cocaine-induced motor disturbances
Source: Transl Psychiatry. 2020 Nov 6;10:381. doi: 10.1038/s41398-020-01050-7 (PMC7648099; doi:10.1038/s41398-020-01050-7)

Figure 1: Overview of the study design and main results

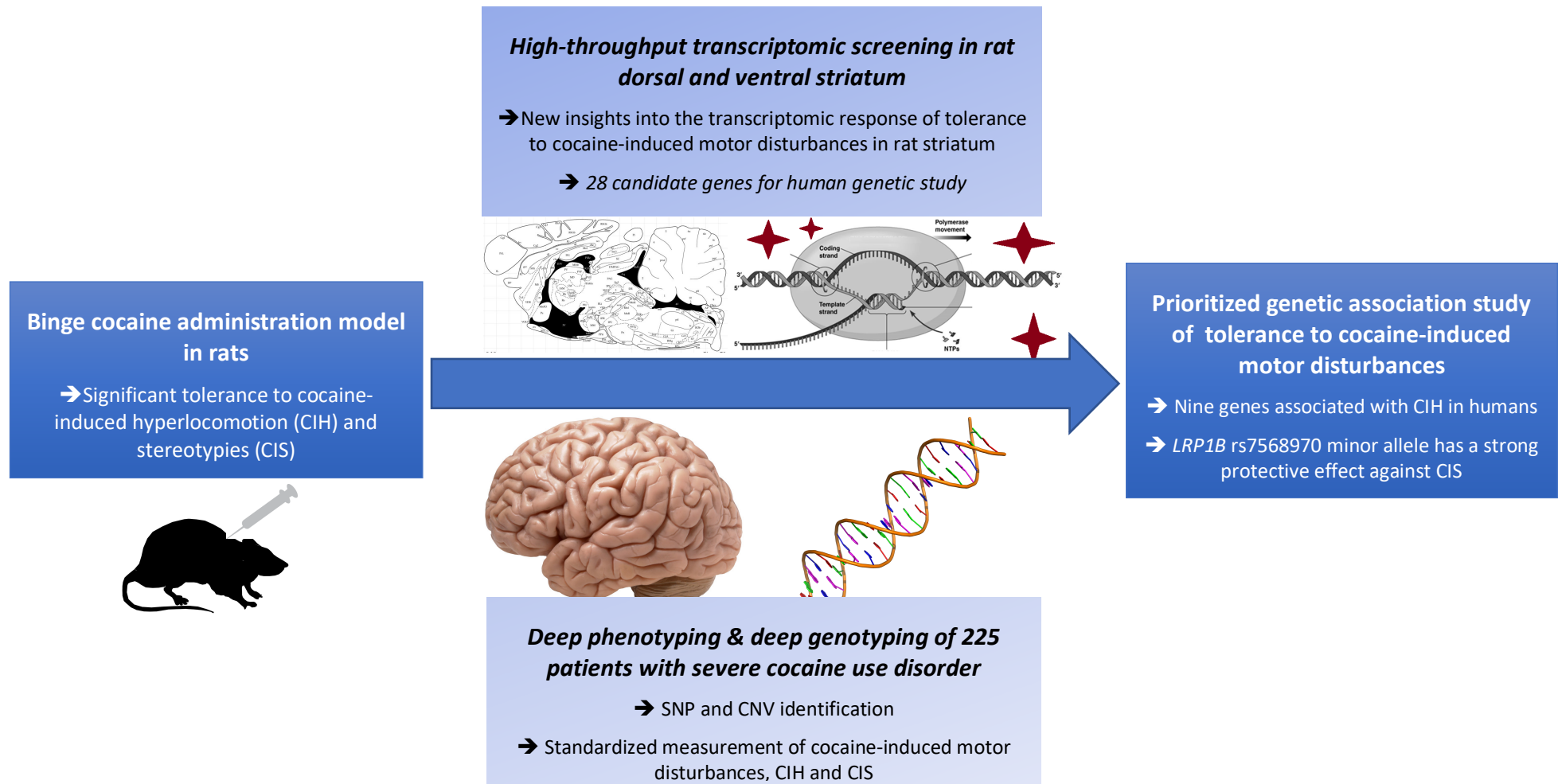

Supplement: Supplementary file 1 — Supplementary Figure 1 [file 41398_2020_1050_MOESM1_ESM.pdf]

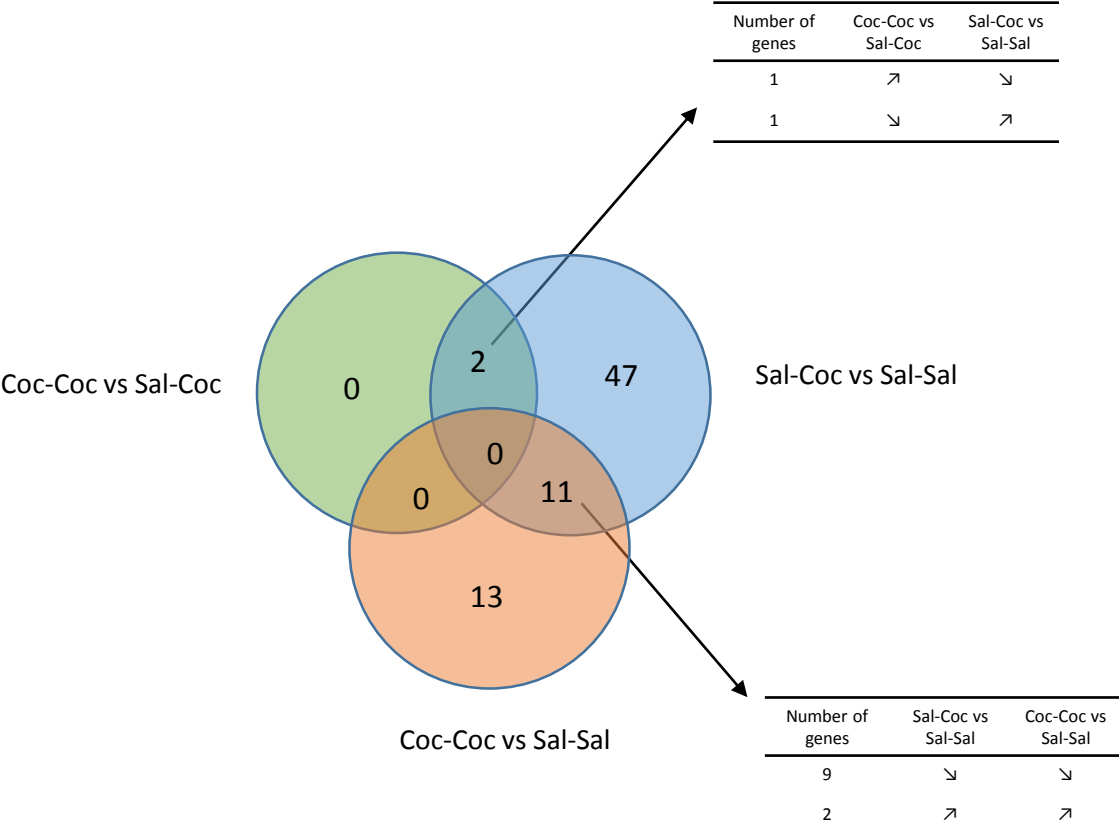

Supplement: Supplementary file 2 — Supplementary Figure 2 [file 41398_2020_1050_MOESM2_ESM.pdf]

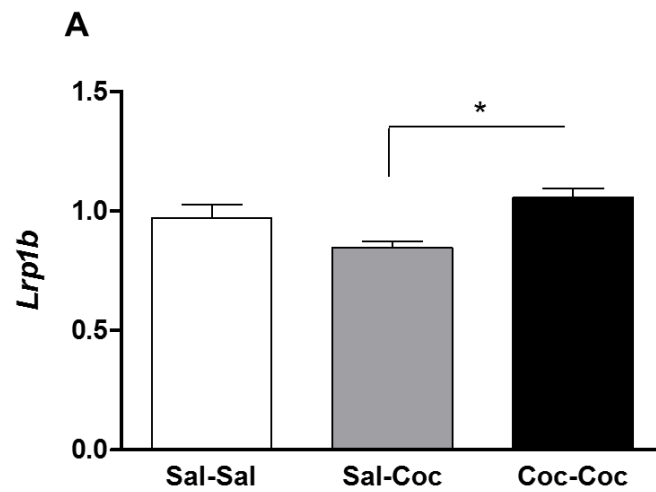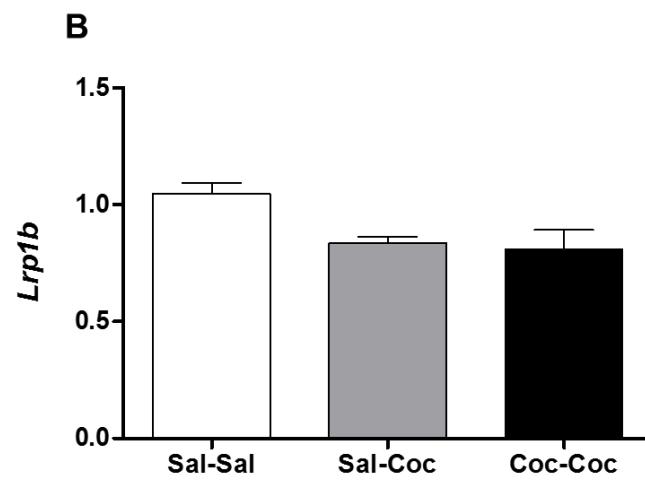

Supplement: Supplementary file 3 — Supplementary Figure 3 [file 41398_2020_1050_MOESM3_ESM.pdf]

Plotted SNPs

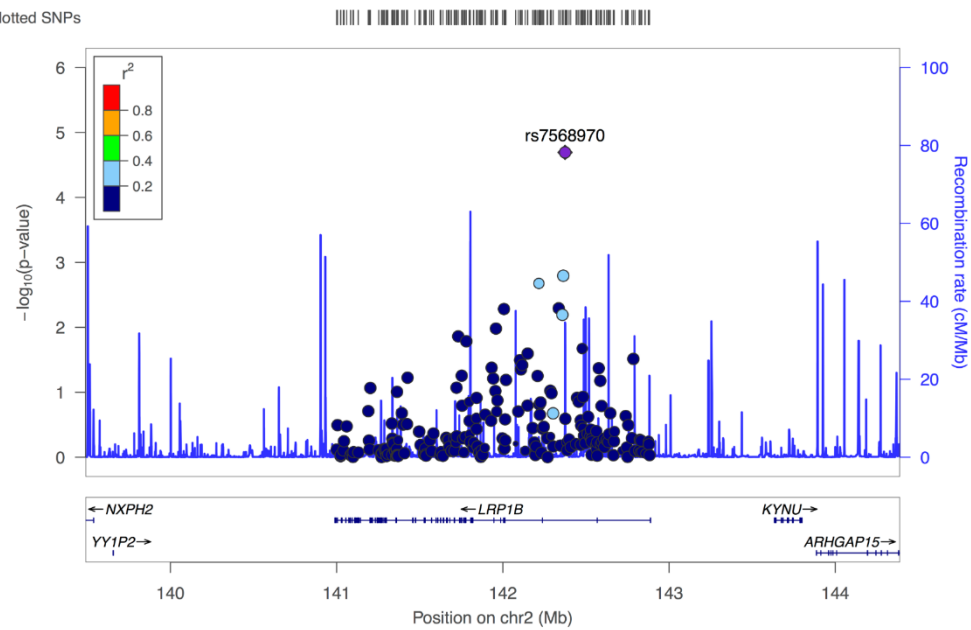

Supplement: Supplementary file 5 — Supplementary Figure 5 [file 41398_2020_1050_MOESM5_ESM.pdf]
