# Supplementary material for: Translational study of the whole transcriptome in rats and genetic polymorphisms in humans identifies LRP1B and VPS13A as key genes involved in tolerance to cocaine-induced motor disturbances
Source: Transl Psychiatry. 2020 Nov 6;10:381. doi: 10.1038/s41398-020-01050-7 (PMC7648099; doi:10.1038/s41398-020-01050-7)
Supplement: Supplementary file 4 — Supplementary Figure 4 [file 41398_2020_1050_MOESM4_ESM.pdf]

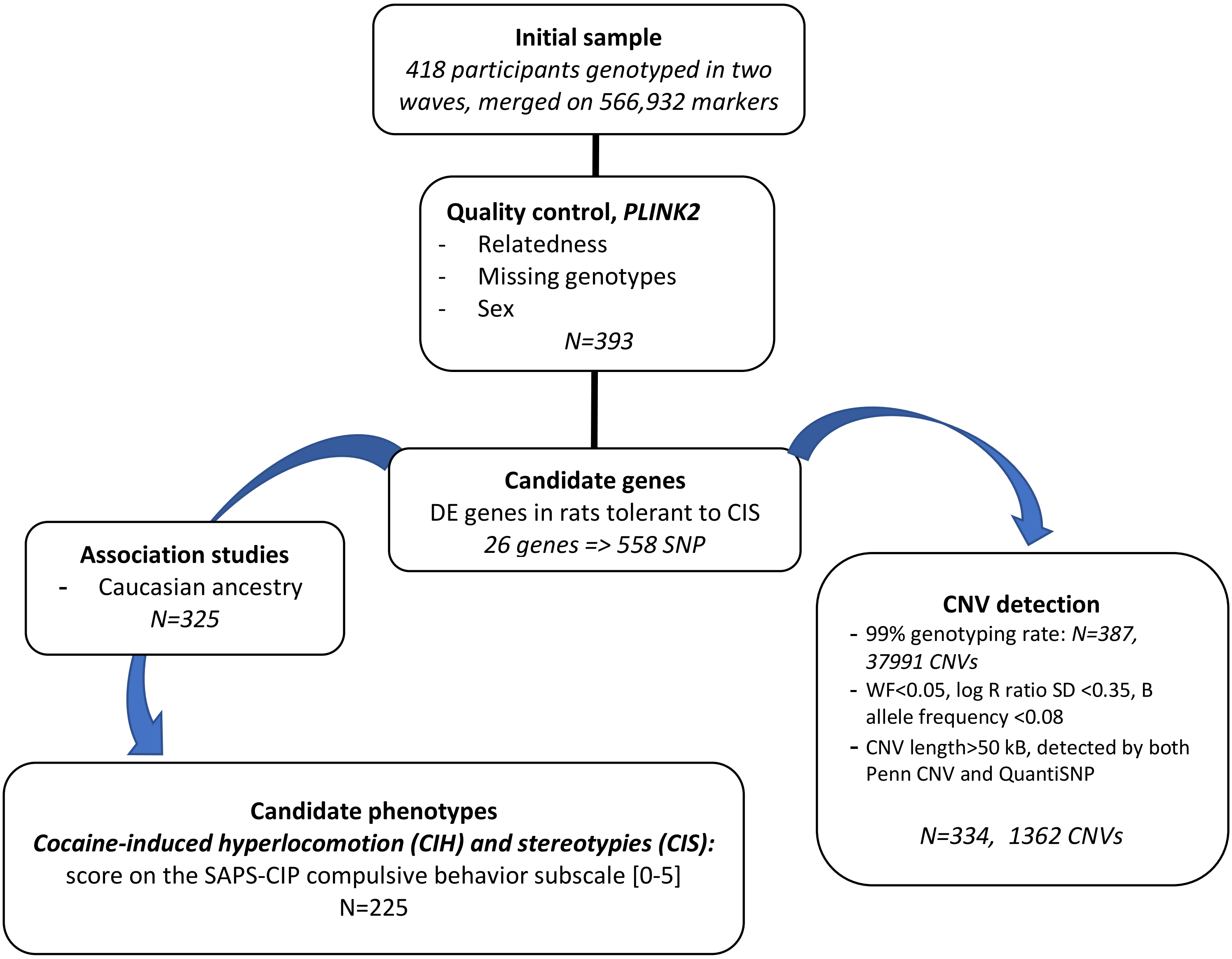
Initial sample
418 participants genotyped in two waves, merged on 566,932 markers
Quality control, PLINK2
- Relatedness
- Missing genotypes
- Sex
N=393
Candidate genes
DE genes in rats tolerant to CIS
26 genes => 558 SNP
Association studies
- Caucasian ancestry
N=325
CNV detection
- 99% genotyping rate: N=387, 37991 CNVs
- WF<0.05, log R ratio SD <0.35, B allele frequency <0.08
- CNV length>50 kB, detected by both Penn CNV and QuantiSNP
N=334, 1362 CNVs
Candidate phenotypes
Cocaine-induced hyperlocomotion (CIH) and stereotypies (CIS):
score on the SAPS-CIP compulsive behavior subscale [0-5]
N=225
