# Supplementary material for: Translational study of the whole transcriptome in rats and genetic polymorphisms in humans identifies LRP1B and VPS13A as key genes involved in tolerance to cocaine-induced motor disturbances
Source: Transl Psychiatry. 2020 Nov 6;10:381. doi: 10.1038/s41398-020-01050-7 (PMC7648099; doi:10.1038/s41398-020-01050-7)
Supplement: Supplementary file 7 — Supplementary Table 2 [file 41398_2020_1050_MOESM7_ESM.docx]

**Supplementary Table 2:** Differentially expressed genes in the ventral and dorsal striatum of Coc-Coc *vs.* Sal-Coc rats and corresponding gene-based associations with cocaine-induced hyperlocomotion (CIH) and stereotypies (CIS) in humans. *significant after Bonferroni correction.

| ***Gene symbol*** | ***Gene description*** | ***Fold change in RNASeq*** | ***Corrected p-value*** | ***Gene symbol in H. Sapiens*** | ***# SNPs after QC*** | ***Gene-based tests raw* p*-value*** | |
| --- | --- | --- | --- | --- | --- | --- | --- |
|  |  |  |  |  |  | **CIH** | **CIS** |
| ***Ventral Striatum*** | | | | | | | |
| *AABR07042733.1* | Cadherin 8 | 1.73 | 2.3E-02 | *CDH8* | 28 | 1 | 1 |
| *Adra2b* | Adrenoceptor Alpha 2B | 0.5 | 2.3E-02 | *ADRA2B* | 0 | 1 | 1 |
| *Ago3* | Argonaute 3 | 2.51 | 2.4E-03 | *AGO3* | 2 | 1 | 1 |
| *Birc6* | Baculoviral IAP Repeat Containing 6 | 1.56 | 7.4E-03 | *BIRC6* | 7 | 6.5E-05* | 0.02603 |
| *Cacna1e* | calcium voltage-gated channel subunit alpha1 E | 1.65 | 1.7E-03 | *CACNA1E* | 34 | 6.5E-05* | 0.02923 |
| *Dgkh* | diacylglycerol kinase eta | 2.69 | 1.38E-11 | *DGKH* | 33 | 1 | 1 |
| *Elavl2* | ELAV Like RNA Binding Protein 2 | 0.61 | 1.02E-02 | *ELAVL2* | 14 | 6.5E-05* | 1 |
| *Fgf2* | fibroblast growth factor 2 | 1.96 | 1.02E-05 | *FGF2* | 5 | 1 | 1 |
| *Glra2* | Glycine Receptor Alpha 2 | 0.59 | 4.83E-02 | *GLRA2* | 0 | 1 | 1 |
| *Grin2b* | Glutamate Ionotropic Receptor NMDA Type Subunit 2B | 1.62 | 4.83E-02 | *GRIN2B* | 45 | 6.5E-05* | 0.02575 |
| *Grm3* | Glutamate Metabotropic Receptor 3 | 1.64 | 1.37E-02 | *GRM3* | 13 | 1 | 0.02923 |
| *LOC100912852* | disks large homolog 5-like | 0.59 | 1.47E-02 | *NA* | | | |
| *Lrp1b* | LDL receptor related protein 1B | 2.69 | 3.6E-06 | *LRP1B* | 220 | 6.50E-05* | 6.50E-05* |
| *Lrrc7* | Leucine Rich Repeat Containing 7 | 1.5 | 3.56E-02 | *LRRC7* | 40 | 6.50E-05* | 1 |
| *Lyst* | Lysosomal Trafficking Regulator | 1.63 | 4.2E-03 | *LYST* | 8 | 1 | 1 |
| *Nefm* | neurofilament, medium polypeptide | 0.61 | 9E-04 | *NEFM* | 2 | 1 | 1 |
| *Nts* | Neurotensin | 0.62 | 2.6E-03 | *NTS* | 1 | 1.40E-05* | 1 |
| *Pvalb* | Parvalbumin | 0.6 | 2.3E-02 | *PVALB* | 5 | 1 | 1 |
| *Rfx3* | regulatory factor X3 | 1.83 | 1.7E-05 | *RFX3* | 11 | 1 | 1 |
| *Scai* | Suppressor Of Cancer Cell Invasion | 1.78 | 7.1E-03 | *SCAI* | 12 | 1 | 0.006752 |
| *Sstr3* | Somatostatin Receptor 3 | 0.44 | 3.61E-02 | *SSTR3* | 1 | 1 | 1 |
| *Taok1* | TAO Kinase 1 (PSK-2, MAP3K16, MARKK) | 1.55 | 1.01E-02 | *TAOK1* | 5 | 1 | 0.09375 |
| *Ubn2* | ubinuclein 2 | 1.9 | 8E-04 | *UBN2* | 4 | 1 | 1 |
| *Vps13a* | Vacuolar Protein Sorting 13 Homolog A | 1.66 | 1.03E-02 | *VPS13A* | 8 | 6.30E-05* | 0.008037 |
| *Zbtb20* | zinc finger and BTB domain containing 20 | 2.57 | 1.63 E-08 | *ZBTB20* | 55 | 6.50E-05* | 1 |
| *Zbtb37* | zinc finger and BTB domain containing 37 | 2.78 | 6E-04 | *ZBTB37* | 1 | 1 | 1 |
| *Zfp871* | zinc finger protein 871 | 1.66 | 4.4E-03 | *NA* | | | |
| ***Dorsal Striatum*** | | | | | | | |
| *L3mbtl1* | Histone Methyl-Lysine Binding Protein 1 | 0.60 | 0.021 | *L3MBTL1* | 5 | 1 | 1 |
| *Lrp1b* | LDL receptor related protein 1B | -0.78 | 2.07E-06 | *LRP1B* | 220 | 6.50E-05* | 6.50E-05* |
