# Supplementary material for: Translational study of the whole transcriptome in rats and genetic polymorphisms in humans identifies LRP1B and VPS13A as key genes involved in tolerance to cocaine-induced motor disturbances
Source: Transl Psychiatry. 2020 Nov 6;10:381. doi: 10.1038/s41398-020-01050-7 (PMC7648099; doi:10.1038/s41398-020-01050-7)
Supplement: Supplementary file 9 — Supplementary Table 4 [file 41398_2020_1050_MOESM9_ESM.pdf]

A.

I. **AGGRESSIVE AND AGITATED BEHAVIOR = 0 1 2 3 4 5**

The subject may behave in an aggressive or agitated manner - often quite unpredictably. He may argue with family, acquaintances, or strangers. He may brandish or use a weapon, or he may attack people or other living things, or he may perform otherwise violent acts.

When you are using cocaine, do you become overly aggressive or agitated. Do you start fights or arguments with friends or with other people? Is this behavior normal for you?

B.

II. **REPETITIVE OR STEREOTYPED BEHAVIOR = 0 1 2 3 4 5**

The subject may engage in a set of repetitive actions or rituals. These actions may involve drug use specifically, or they may be more general. For example, "geeking" is a behavior seen in cocaine users where the individual engages in compulsive foraging for remnants of cocaine in the vicinity of where it was used. Also, subjects may develop a ritual while using that they perform over and over again. Or, subjects may exhibit repetitive finger tapping, picking or tugging at hair or clothing, humming, snorting, etc.

When you are using cocaine, is there anything that you do a lot? Do you have things that you will do over and over again? Do you have the feeling that you have to do certain things, like getting up to check the windows or the door locks. Do you look in the mirror for a long period of time? How much time do you spend doing theses things? Does it ever seem to you that there is cocaine scattered around, or do you look everywhere to make sure there is no more?

C.

		Mean (SD) or N(%)
Age		38 (9)
Gender	*Men*	178 (79%)
	Women	47 (21%)
Homelessness ≥3 months		89 (29%)
At least one lifetime SA		115 (38%)
Number of cigarettes smoked / day		18 (11)
3 comorbid lifetime SUDs or more (except tobacco)		257 (44%)
Lifetime SUD diagnoses	*Opiates*	129 (42%)
	Alcohol	224 (75%)
	Cannabis	243 (81%)
	Sedatives	170 (56%)
Age at onset of cocaine use disorder (years)		27 (8)
Weekly cocaine use during self-reported worst period of CUD (daily *vs.* non-daily)		161 (72%)
CIS		2.3 (1.5)
	Median (IQR)	3 (1-3)
	≥2 (clinical significance threshold)	159 (71%)
CIH		1.6 (1.8)
	Median (IQR)	1 (0-3)
	≥2 (clinical significance threshold)	104 (46%)
