# Supplementary material for: Translational study of the whole transcriptome in rats and genetic polymorphisms in humans identifies LRP1B and VPS13A as key genes involved in tolerance to cocaine-induced motor disturbances
Source: Transl Psychiatry. 2020 Nov 6;10:381. doi: 10.1038/s41398-020-01050-7 (PMC7648099; doi:10.1038/s41398-020-01050-7)
Supplement: Supplementary file 11 — Supplementary materials [file 41398_2020_1050_MOESM11_ESM.docx]

**Manuscript “Translational study of the whole transcriptome in rats and genetic polymorphisms in humans identify LRP1B and VPS13A as key genes in tolerance to cocaine-induced motor disturbances” – *supplementary methods file***

**Chemicals.** Cocaine hydrochloride (Francopia, Anthony, France) was dissolved in sterile saline solution (0.9% (w/v) NaCl). Rats received 20 mg/kg cocaine in 1 ml/kg of body weight by intraperitoneal (i.p.) route.

1. *Clinical study*

**Sample selection and clinical assessment:** Eligible participants were excluded if they had severe cognitive impairment or insufficient mastery of the French language, if they had no social insurance, and if they were under compulsory treatment. The *Scale for Assessment of Positive Symptoms – Cocaine-Induced Psychosis (SAPS-CIP)* was developed in order to assess a broad range of hallucinations, delusions and compulsive behaviors that can accompany cocaine use by rating them on a 0- to 5-point scale according to their intensity (1). It was translated to French by our group (2) using translation and back-translation procedures. A broad range of sociodemographic and clinical variables was also collected, especially regarding the course of cocaine use and the comorbidity with non-cocaine SUDs over the lifetime (Supplementary Table 3B).

**Biological sampling:** Participants’ DNA purity assessment followed the procedures described by the Centre National de Génomique using a NanoDrop spectrophotometer and PicoGreen assay.

**Genetic analyses — single nucleotide polymorphisms (SNPs):** At the whole-sample (N=394) and genome-wide levels, we excluded markers and individuals with > 2% genotype missingness, checked for sex discrepancy, selected SNPs [SNVs with minor allele frequency (MAF) values ≥ 5%], and excluded variants that deviated from Hardy-Weinberg Equilibrium at *p* < 10^-6^, leaving 393 individuals and 260,853 markers. A search for cryptic relatedness by identity-by-descent, using a piHat threshold of 0.2 (2^nd^ degree relatedness), further identified 13 pairs of related individuals; the individual with the lower genotyping rate was excluded from each of these pairs. For the genetic association study, we further excluded 43 individuals of non-Caucasian ancestry according to the distribution of SNPs in our samples, which were merged and compared with the latest version of the 1000 genomes project (1000G) panel, based on visual inspection of principal component analysis (N=2502 from five superpopulations), according to a standard procedure (3). Among the differentially expressed genes (DEGs) identified in the preclinical study, two genes, *LOC100912852* and *Zfp871*, do not occur in the human genome, and *LRP1B* was identified in both brain areas.

**Genetic analyses — copy number variants (CNVs):** Two detection programs, *PennCNV* (4) and *QuantiSNP* (5), optimized in a combined algorithm, were used to perform a thorough quality check (see details in Fig. 3). These programs have different sensitivity and specificity. The aim of this analysis was to identify additional variants that would be relevant to fuel further *in vitro* and *in vivo* functional analyses. It was thus performed at both the genome-wide and sample-wide levels. We considered only individuals and CNVs meeting stringent control criteria (Fig. 3).

1. *Data analysis*

**Phenotype assessment***.* Continuous variables were described by means (standard deviation, SD) or medians (interquartile range, IQR) depending on their distribution, and discrete variables by absolute counts and frequencies. Nonparametric tests were used to identify the clinical and sociodemographic factors associated with cocaine-induced stereotypies (CIS) for further incorporation into the ordinal regression model.

**Human genetics**: **in silico *functional analyses*** were performed online on November 20^th^ 2018 using the *Functional Mapping and Annotation of Genome-Wide Association Studies (FUMA)* platform (7), which allows for an all-at-once, yet fully customizable functional assessment from 18 biological data repositories and tools. *FUMA* was searched for variant type and location, gene function assessment/pathway enrichment, eQTL and mQTL mapping in the brain, transcription factor binding and chromatin conformation. The *3D* *Genome browser* (8) was used, in addition, to identify potential distant regulatory effects according to the topologically associating domains (9).

**References**

1. Cubells JF, Feinn R, Pearson D, Burda J, Tang Y, Farrer LA, *et al.* (2005): Rating the severity and character of transient cocaine-induced delusions and hallucinations with a new instrument, the Scale for Assessment of Positive Symptoms for Cocaine-Induced Psychosis (SAPS-CIP). *Drug Alcohol Depend*. 80: 23–33.

2. Vorspan F, Brousse G, Bloch V, Bellais L, Romo L, Guillem E, *et al.* (2012): Cocaine-induced psychotic symptoms in French cocaine addicts. *Psychiatry Res*. 200: 1074–1076.

3. Marees AT, Kluiver H de, Stringer S, Vorspan F, Curis E, Marie‐Claire C, Derks EM (2018): A tutorial on conducting genome-wide association studies: Quality control and statistical analysis. *International Journal of Methods in Psychiatric Research*. 27: e1608-17.

4. Wang K, Li M, Hadley D, Liu R, Glessner J, Grant SFA, *et al.* (2007): PennCNV: An integrated hidden Markov model designed for high-resolution copy number variation detection in whole-genome SNP genotyping data. *Genome Res*. 17: 1665–1674.

5. Colella S, Yau C, Taylor JM, Mirza G, Butler H, Clouston P, *et al.* (2007): QuantiSNP: an Objective Bayes Hidden-Markov Model to detect and accurately map copy number variation using SNP genotyping data. *Nucleic Acids Res*. 35: 2013–2025.

6. Li H, Handsaker B, Wysoker A, Fennell T, Ruan J, Homer N, *et al.* (2009): The Sequence Alignment/Map format and SAMtools. *Bioinformatics*. 25: 2078–2079.

7. Watanabe K, Taskesen E, Bochoven A van, Posthuma D (2017): Functional mapping and annotation of genetic associations with FUMA. *Nature Communications*. 8: 1826.

8. Wang Y, Song F, Zhang B, Zhang L, Xu J, Kuang D, *et al.* (2018): The 3D Genome Browser: a web-based browser for visualizing 3D genome organization and long-range chromatin interactions. *Genome Biology*. 19: 151.

9. Yu W, He B, Tan K (2017): Identifying topologically associating domains and subdomains by Gaussian Mixture model And Proportion test. *Nature Communications*. 8: 535.
